# Healthcare needs of the Muslim patient community in the undergraduate medical curriculum – Are we there?

**DOI:** 10.12669/pjms.35.3.861

**Published:** 2019

**Authors:** Hameedul Haq, Rehan Ahmed Khan, Raheela Yasmin

**Affiliations:** 1 *Dr. Hameedul Haq, MBBS, MHPE. Department of Health Professions Education and Research, Peshawar Medical College, Peshawar, Pakistan*; 2 *Prof. Dr. Rehan Ahmed Khan, MBBS, FCPS, FRCS, JM-HPE, MSc HPE. Department of Surgery, Islamic International Medical College, Riphah International University, Islamabad, Pakistan*; 3 *Prof. Dr. Raheela Yasmin, BDS, DCPS-HPE, JMHPE, MHPE, PhD-HPE Scholar. Riphah Academy of Research and Education, Islamic International Medical College, Riphah International University, Islamabad, Pakistan*

**Keywords:** Curriculum planning, Muslim, Patient needs, Content inclusion, Delphi method

## Abstract

**Objective:**

Muslim patients have a unique set of healthcare needs that are related to their faith. These are generally not formally addressed in the medical curricula. The study aimed to recommend additional content that would better tailor the undergraduate curriculum to cater to the needs of this large cohort – Muslim patients. This is with the expectation that patients would have their faith-related health queries resolved by healthcare providers.

**Methods:**

A quantitative descriptive survey design was adopted. A 46-item questionnaire formulated through a literature review was put forth to experts using the Delphi Technique. Experts were selected based on having an academic rank of associate professor and above or medical education credentials. Three iterative rounds were conducted for exploring consensus over a period of five months. Panel agreement of >70% was the criteria for inclusion.

**Results:**

Items were categorized under 7 subject themes: Medicine, Psychiatry, Surgery, Gynecology, Obstetrics, Medical Ethics, and Islamic Studies. Consensus was eventually reached for 41 out of 46 items. These topics included but were not limited to “The Muslim patient in Ramadan to: fast or not to fast?” and “Muslim women and decision-making on pregnancy termination”.

**Conclusion:**

The study suggested that the topics proposed herein were in fact legitimate faith-related healthcare needs of Muslim patients. Their inclusion would add value to the undergraduate medical curriculum and would train practitioners to improve patient outcomes more holistically.

## INTRODUCTION

Muslims comprise almost one-fourth of the world population.^1^ The Islamic faith brings along a code of life that affects its adherents’ everyday decisions in very tangible ways, which encompasses healthcare as well. Consequently, a multitude of healthcare related scenarios exist for which religious input is necessary. Some examples include the use of insulin during fasting, optional abortion, cosmetic surgery, matters related to euthanasia, and urinary tract surgery and its post-op implications regarding the maintenance of a state of ritual purity (ablution).^2^-^6^

These decisions lie in an area of overlap between the patient, doctor, and religious scholar who must all be part of a validated process. Currently, patients are referred to a scholar who should (but may not) consult a doctor before answering faith-related medical questions. Alternatively, answers are sought from the doctor who again should (but may not) consult a scholar before answering medical questions that require faith-related knowledge. Both reportedly take ‘religiously-informed clinical decisions’ based on personal knowledge, asking of peers, or browsing online and offline resources. Irrespective of the strategy used to tackle clinical scenarios pertaining to these needs, a more reliable formula is required to ensure better patient outcomes. Dependable solutions are not always reached in resolving these serious queries due to the lack of verifiability in the above-mentioned ‘default’ mechanism.

Bridging this particular knowledge gap will equip future graduates for faith-related clinical scenarios, before they end up struggling to learn it themselves. It is seen that issues pertaining to other religious communities have successfully gone through the rigorous process of being incorporated in the medical curricula. For example, there is literature on managing Jehovah’s Witnesses patients who refuse blood transfusions based on their religious beliefs, even though this particular Christian denomination comprises less than 0.1% of global population.^7^ Hence, training doctors regarding similar issues in the Islamic context could be an efficient approach in addressing the lack of well-grounded, faith-related medical knowledge required to manage these needs. R. M. Harden, a world leader in medical education, states: “Increasing emphasis is being placed on having an *authentic* curriculum – a curriculum where the content is more closely related to the work of the practicing doctor”.^8^ This study aimed to propose the most relevant topics in each subject that can be added to the undergraduate curricula.

## METHODS

The study had two objectives. First, to identify the needs of Muslim patients and second, to explore consensus among experts for addressing them in the undergraduate medical curriculum. It was a quantitative descriptive survey research conducted at Riphah International University with non-probability purposive sampling of the faculty population and its health professions education graduates. An email was sent out with a brief introduction of the study and a link to the online questionnaire for those consenting to participate. It was followed by reminders at weekly intervals for three iterative questionnaire rounds conducted over a period of five months.

**Fig.1:**
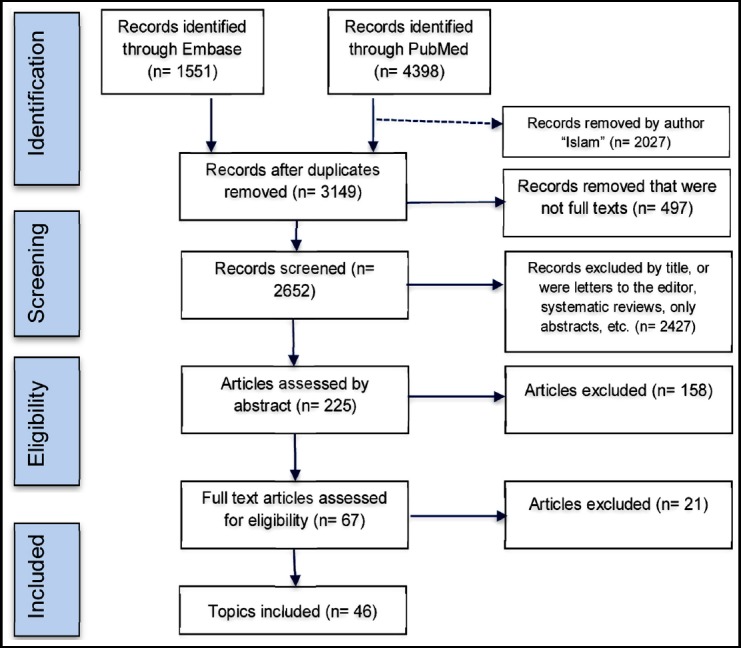
PRISMA model. Preferred Reporting Items for Systematic Review and Meta-Analysis (PRISMA) flowchart for the keywords used in the literature review: Muslim AND Patient.

To identify topics related to Muslim patients, a literature search was conducted within online and offline medical databases, including PubMed, Embase, and the Federation of Islamic Medical Associations annual publications. Consequently, a list of 46 topics was developed for the study’s questionnaire. The process of selecting these is shown in the PRISMA chart below (Fig.1). The rarity of available related research prevented any selection bias because the resultant list included *all* the relevant topics found.

Secondly, to explore consensus among experts, the Delphi technique was employed.^9^ The criterion for expert selection was an academic rank of associate professor and above or medical education credentials. They included both clinicians and teachers, given their expertise in the curricular needs of students by virtue of their overlapping roles in most healthcare institutions. A panel of 27 experts was engaged over a period of five months to ponder the inclusion of listed topics in the curriculum. This entailed sending out three iterative rounds of questions via email, where the results of each previous round were anonymously shared with the panel for the sake of conducting a subsequent round. This allows informed participants the opportunity to modify their responses in light of the whole panel’s opinion, until an overall consensus could be established.

For the sake of analyzing stability in responses, all of the topics from Round One were again included on the Round-2 questionnaire, including those topics that had consensus. The general definitions for “consensus” are considered to be anywhere between 51% and 80% agreement. The criteria in this study was set at the *typical* definition of 70%.^10^ This means that 70% of the experts rated an item 4 or 5 on the Likert scale i.e. to “Include” or “Definitely Include” the topic. Three rounds were conducted until consensus was evident for an overwhelming majority of topics.

Finally, an open-ended question was *conditionally* added for whenever a topic was rated as 1 or 5 on the Likert scale (i.e. “Definitely Include” or “Definitely Exclude”) where the expert was asked to provide a reason for their strong choice.

## RESULTS

A list of 46 potential needs of Muslim patients was developed through the literature search that subsequently formed the study’s questionnaire. Using the Delphi technique, consensus of experts eventually developed on the inclusion of a number of these identified topics in the undergraduate curriculum. The stability of responses from round to round was established using the Chi-Square Test.^11^ The consensus percentages for each item from Round 1 were taken as the *observed range*. The consensus percentages from Round 2 were taken as the *expected range*. The P-value calculated was 0.0008, demonstrating a very stable set of response data.

Table-I lists by discipline the needs/topics identified within in literature search that were presented to the expert panel. The final percentages of agreement on inclusion by the last round are displayed in the third column. Comprising of 46 topics in total, 27 topics were agreed upon in the first round. A further 10 out of the remaining 19 topics were included in the second round. And finally, four more topics out of the remaining nine were selected for inclusion in the third round. The remaining five topics were excluded.

**Table-I:** Questionnaire Topics.

Discipline	Topics	Consensus %
Medicine	1. *Epilepsy: catering for Muslims’ beliefs about causes and treatment.*	63.0
	2. *Prevention of chronic hepatitis B and C: role of Islamic teachings.*^12^	88.9
	3. *The halal status of medicinal ingredients and excipients.*^13^	81.5
	4. Islamic perspectives on clinical intervention near the end-of-life.	88.9
	5. *Content of spiritual counseling for Muslim cancer patients.*^14^	85.2
	6. Brain death from the Islamic viewpoint.	88.9
	7. Approaching the Muslim patient: cultural competence for non-Muslim practitioners.	81.5
	8. Seeking remedy or abstaining from therapy: a Muslim patient’s approach.	74.1
Psychiatry	1. *Alcoholism and drug dependence in the Muslim context.*	77.8
	2. *Management of depression in Muslim patients - using adapted behavioral activation.*^15^	88.9
	3.Psychotherapeutic needs of religious Muslim patients.	85.2
	4. *’My spouse is possessed by a jinn’: studying transcultural mental health.*	59.3
	5. *Addressing needs of suicidal Muslim patients.*	70.4
Surgery	1. *Grafting and surgical products: Islamic limitations.*	92.6
	2. Aesthetic and cosmetic surgery in Islam.	66.7
	3. Orthopedic problems and performing the Muslim prayer.	85.2
	4. Urinary tract surgery and its impact on Islamic rituals.	81.5
	5. Stoma-specific fatwas in acts of worship for the Muslim patient.	74.1
	6. Organ transplantation in the Islamic faith.	85.2
	7. Organ donation in the Muslim religion.	88.9
Gynecology	1. Practicing sexual health medicine: challenges in the Muslim world.	74.1
	2. *Disorders of sex development and gender reassignment in Islam.*^16^	77.8
	3. *Islamic religious beliefs: issues in gynecological practice.*	85.2
	4. *Abnormal vaginal bleeding and its effect on Muslim women’s rituals.*	85.2
	5. *Coping with infertility: the resource of religion and spirituality.*^17^	70.4
	6. Contraception: Family planning with the Islamic patient.	96.3
	7. Religion and assisted reproductive technology.	81.4
	8. *Hymenoplasty: Islamic ethico-legal views*.	51.8
Obstetrics	1. *Milk kinship in Islam: an obstacle in donor human milk for preterm infants?*	74.1
	2. *Prevention and management of genetic disorders: ethical religious considerations.*^18^	77.8
	3. *The role of faith in antenatal screening of congenital anomalies.*^19^	77.8
	4. *Fetal sex identification and pre-selection in Islam.*^20^	85.2
	5. Muslim women and decision-making on pregnancy termination.	81.5
	6. Abortion of rape-related impregnation in Muslim populations.	81.5
Medical Ethics	1. *Breaking bad news: practical guidelines for Muslim practitioners.*^21^	96.3
	2. Understanding faith considerations when caring for bereaved Muslims.	88.9
	3. Physician-assisted suicide and euthanasia in Muslim patients.	81.5
	4. Do not resuscitate: the Islamic patient.	70.4
	5. Patient rights: an Islamic perspective.	96.3
	6. ’Medical futility’ as applied in Islam.	62.9
	7. “Necessity” in Islamic medical ethical assessment.	88.9
Islamic Studies	1. The Muslim patient in Ramadan: to fast or not to fast?	96.3
	2. *Post-stroke issues for Muslim patients in maintaining the daily prayers.*^22^	92.6
	3. Religious needs of hospitalized Muslim patients.	92.6
	4. *Spiritual needs assessment tool for the Muslim patient.*	74.1
	5. *Spiritual care and its effect on healing in Islamic patients.*^23^	88.9

Key:-* Strikethrough: topics that remained excluded after the last Delphi round.

** *Italicized*: additional topics that were newly proposed through the study.

### Themes in “Definitely” Choice

The study asked those experts who chose to “Definitely Include” or “Definitely Exclude” a topic i.e. rated it as a 1 or 5 on the Likert scale, to explain why they made that choice. Overwhelmingly topics were rated highly for *inclusion*. Upon analysis of all the panels’ explanations, three major reasons could be extracted:

### Reason 1

The most frequent reason provided for a topic to be included was that the item was a *common* problem. Practitioners encountered the issue frequently, not knowing how to address it.

### Reason 2

Another reason for which inclusion percentages were high was that an item was perceived to be a *high stakes* issue. Leaving it unaddressed could cost lives and precious resources.

### Reason 3

The idea that something was being *neglected* altogether was another popular reason for an item’s inclusion since there were no recognizable attempts at addressing the need.

## DISCUSSION

One of the major challenges of this study was nonexistence of literature dealing directly and holistically with this area. As a result, it warranted an exhaustive initial literature search to arrive at the latter compilation of topics/needs unique to Muslim patients. The limited number of individual articles dealing with clinical issues pertaining to this cohort were brought together, each of which then served as a resource for building the final list.

Endeavors have been made at devising segments in the undergraduate medical curriculum to cater for health issues related to the Islamic faith. One of those – known as the Islamic Input in the Medical Program (IIMP) or Islamic Input in the Medical Curriculum (IIMC) – was pioneered by the Kulliyah of Medicine at the International Islamic University of Malaysia in 1997. Various forms of it have been incorporated at medical schools around the world, including the Institute of Medicine at Universiti Brunei Darussalam, Brunei, the Universiti Sains Islam, Malaysia, Riphah International University, Pakistan, and 14 other schools that are members of FOKI (Forum Kedokteran Islam Indonesia).^24^

This would indicate that a significant amount of work has been carried out in this area and that the IIMP might already address numerous faith-related needs of Muslim patients. It is pertinent to mention here the difference of approach in the work that has been carried out. The IIMP was developed as part of a broader vision to Islamize knowledge and consists of two separate but closely related components: Islamization and Medical Jurisprudence. Islamization deals with putting medicine in an Islamic context in terms of epistemology, values and attitudes by bridging the dichotomy between traditional Islamic sciences and the medical sciences. Medical Jurisprudence deals with issues of application of the Law (*Sharia*) from a medical perspective.^25^

In contrast, the approach of this study was to focus on the needs of Muslim patients in order to identify potential content for the undergraduate medical curriculum within both of the above-mentioned components. That being said, the generality of topics identified fall under the Medical Jurisprudence category of the IIMP, which comprises modules on the jurisprudence of acts of worship, physiological conditions, pathological conditions, and reproductive and genetic technology. Nevertheless, this study identified additional topics within each subject category, which were not part of earlier curricula. Major distinguishing factors here include the process and technique employed for this proposal. Not only was a thorough literature search undertaken to identify the needs of the population, but the critical consensus of experts was also sought.

### Limitations of the study

1. Structural quality of the items on the questionnaire.
2. Uneven representation in the specialty of experts.

## CONCLUSION

There is a neglected spectrum of medical issues, which are faith-related, that exist for Muslim patients. These are generally not catered for by the existing curricula in most medical schools, despite being the need of their local populations. Hence 46 such topics were identified from available literature that could help in addressing this inadequacy. A panel of experts was engaged to seek consensus on these topics and by the end of the process it was agreed that 41 of them are in fact health needs of Muslim patients. On that account, they merit inclusion on the undergraduate medical school curricula in nation-states with significant Muslim populations.

## RECOMMENDATIONS

Moving forward, the contextual needs of patients should be sought when reforming curricula. At present, the agreed upon topics must find a way to the curriculum for future healthcare practitioners. For those who are already part of the workforce, literature on these topics shall be identified to bridge their knowledge gap, as part of continuing professional development.

### Author’s Contribution

**HH:** Conception, data collection and analysis, manuscript writing.

**RAK:** Design, final editing of manuscript.

**RY:** Critical review.
